# Synthesis of an Al-Based Composite Reinforced by Multi-Phase ZrB_2_, Al_3_BC and Al_2_O_3_ with Good Mechanical and Thermal Properties at Elevated Temperature

**DOI:** 10.3390/ma13184048

**Published:** 2020-09-12

**Authors:** Yihan Bian, Tong Gao, Yongfeng Zhao, Guiliang Liu, Xiangfa Liu

**Affiliations:** 1 Key Laboratory for Liquid-Solid Structural Evolution and Processing of Materials (Ministry of Education), Shandong University, Jingshi Road 17923, Jinan 250061, China; yhbian@yeah.net (Y.B.); zyf200900150317@163.com (Y.Z.); xfliu@sdu.edu.cn (X.L.); 2 Shandong Mai Ao Jing Advanced Materials Co. Ltd., Linyi 276000, China; 18678391822@163.com

**Keywords:** Al composite, multiphase, elevated temperature properties

## Abstract

To synthesize Al composite with high strength at elevated temperature, high modulus and thermal stability, ZrB_2_, Al_3_BC and Al_2_O_3_ particles have been chosen as reinforcements simultaneously. A (9.2 wt.% ZrB_2_ + 5.6 wt.% Al_3_BC + 5.5 wt.% Al_2_O_3_)/Al composite has been prepared, and the in-situ synthesized particles are nano-sized. Mechanical property tests reveal that the nanoparticles exhibit a remarkable synergistic enhancement effect. The elasticity modulus of the composite is 89 GPa, and the ultimate tensile strengths at 25 °C and 350 °C can be as high as 371 MPa and 154 MPa, respectively.

## 1. Introduction

Aluminum matrix composites (AMCs), as a green engineering material, are widely used in the aerospace, transportation and electronic information fields due to the low density and high specific strength [1,2,3]. In some special conditions, such as aircraft engine compartments and automotive pistons, the working temperature can be up to 300 °C, therefore high strength and thermal stability of the materials at elevated temperatures are critical [4]. Currently, the most widely used heat-resistant Al alloys, e.g., 2618 (Al–Cu–Mg–Ni–Fe) and 2219 (Al–Cu), are mainly enhanced by solution strengthening by θ’ precipitates [5,6]. However, their properties drop sharply with temperatures exceeding 300 °C due to the coarsening of nano-precipitates or the harmful phase transition of metastable intermetallic compounds [7]. Besides, the rigidity sometimes also fails to meet the requirement. The ceramic reinforced particles in AMCs commonly have high melting points and good thermal stability, which provides great potential for improving the high temperature performance of Al alloys [8].

The mechanical and thermal properties of AMCs are mainly determined by the type, size and distribution of the reinforced particles as well as their interfaces with the matrix [9]. Ceramic particulates such as oxides (Al_2_O_3_, SiO_2_, ZrO_2_, etc.), carbides (Al_4_C_3_, SiC, TiC, etc.), nitrides (AlN, Si_3_N_4_, etc.) and borides (TiB_2_, ZrB_2_, etc.) are commonly utilized as enhancements based on their distinctive characteristics [10,11,12,13]. For instance, Al_2_O_3_ has a low density and high ultimate strength, and is widely used in the industrial production of high-strength materials [14]. SiC with high hardness can significantly improve the wear resistance of the composite, while AlN has been reported as beneficial for the strength of the Al matrix at high temperatures due to its special distribution [15]. The in-situ formed ZrB_2_ particles have been reported as helpful for the improvement of the mechanical properties of the AA5052 alloy [16]. Besides, in the previous study of our group, Al_3_BC particles with high modulus (326 GPa) and low density (2.83 g/cm^3^) have been successfully in-situ synthesized, which led to significant enhancement of the tensile strength of 6061Al matrix at 300 °C [17].

Compared with the AMCs reinforced with a single particle, utilizing the heterogeneous strengthening effects of multiphase particles to achieve good comprehensive performance of the composite has attracted attention in recent years [18,19,20]. For the design of multiphase reinforced particles, on the one hand, the chosen particles need to match with each other, in order to achieve the desired comprehensive properties of the composites [21]. On the other hand, it is necessary to avoid the generation of unexpected or harmful phases during the chemical reaction process [22]. For example, the in-situ synthesis of Ti-containing particles may be accompanied by flaky and coarse TiAl_3_ particles in the Al–TiN system [23], and B-containing particles easily form harmful AlB_2_ and AlB_12_ together with the desired Al_2_O_3_ particles [24], finally resulting in stress concentration and matrix fracture of the composites.

According to our previous work [25], ZrB_2_ and Al_2_O_3_ have been proven to be useful reinforcements to improve the high-temperature performance of Al composite. Therefore, in order to enhance the modulus of the composite simultaneously and to avoid the generation of harmful phases, the Al_3_BC particles with high modulus were in-situ introduced. An aluminum composite (ZrB_2_ + Al_3_BC + Al_2_O_3_)/Al with high strength, good thermal stability and high modulus was designed, and related analysis has been carried out.

## 2. Materials and Methods

**Figure 1:**
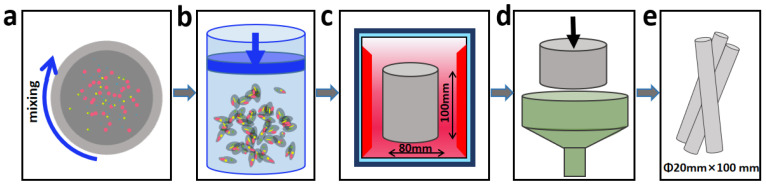
Schematic diagram of composite preparation process: (**a**) mixing the powders; (**b**) pressing the powders into an ingot; (**c**) heating the ingot to 800 °C; (**d**) extruding the heated ingot; (**e**) the achieved rods after extrusion.

The materials used in this paper include commercial pure Al powders (99.7 wt.%; ~2 μm, Qinhuangdao ENO High–Tech Material Development Co. Ltd., Qinhuangdao, China), commercial pure ZrO_2_ powders (99.9 wt.%; ~50 nm, Qinhuangdao ENO High–Tech Material Development Co. Ltd., Qinhuangdao, China), and commercial carbon and boron plasmid powders (97 wt.%; ~0.5 μm; provided by Shandong Mai Ao Jing Advanced Materials Co. Ltd., Linyi, China). Figure 1 is a schematic diagram of the preparation procedure. As shown in Figure 1a, all the powders are weighed and mixed completely, and then compressed into an ingot with the size of Φ 80 mm × 100 mm at 280 MPa in a cold isostatic press machine whose type is LDJ200/500–380YS (Figure 1b). The composite containing 9.2 wt.% ZrB_2_, 5.6 wt.% Al_3_BC and 5.5 wt.% Al_2_O_3_ was fabricated through the liquid–solid reaction at 800 °C for 1 h in a vacuum furnace (Figure 1c). Finally, the (ZrB_2_ + Al_3_BC + Al_2_O_3_)/Al composite was extruded into a rod shape to eliminate voids (Figure 1d). The extruded composite rods are shown in Figure 1e, with the diameter of 20 mm. For comparison, pure aluminum rods were also produced through the above process.

The microstructure of the composite was characterized by the field emission scanning electron microscope (FESEM, JSM–7800F, Japan Electron Optics Laboratory Co. Ltd., Tokyo, Japan) operated at 15 kV, linked with an energy dispersive X-ray spectrometry (EDS) attachment. The X-ray diffraction (XRD) was performed with the Rigaku D/max–rB diffractometer (Rigaku, Tokyo, Japan) (Cu Ka, 40 kV, 100 mA) to identify the ZrB_2_, Al_2_O_3_ and Al_3_BC phase. The morphology and distribution of the nanoparticles were observed using a transmission electron microscope (TEM, FEI Talos F200X, Hillsboro, OR, USA), and the high-resolution transmission electron microscopy (HRTEM) analysis was carried out to obtain lattice images of the nanoparticles.

The hardness of the composite and pure Al was tested using a digital Brinell hardness tester (HBST–3000AET, Hua Yu Zhong Xin Co. Ltd., Yantai, China). The loading force was 2452 N and dwell time was 60 s and the obtained value was an average of at least five measurements. The solid material dynamic elasticity modulus tester (IET–01, Netzsch, Selb, Germany) was used to investigate the elasticity modulus. The thermal stability of the composite and pure Al was measured under Argon gas using a high temperature dilatometer at a heating rate of 10 K/min. The electronic all-purpose test machine (CMT700) was used to conduct tensile tests at room temperature (25 °C) and high temperature (250 °C, 350°C and 450 °C) with a tensile rate of 2 mm/min. For high temperature tests, the samples were heated to the corresponding temperature and kept for 30 min before force loading [26].

## 3. Results and Discussion

**Figure 2:**
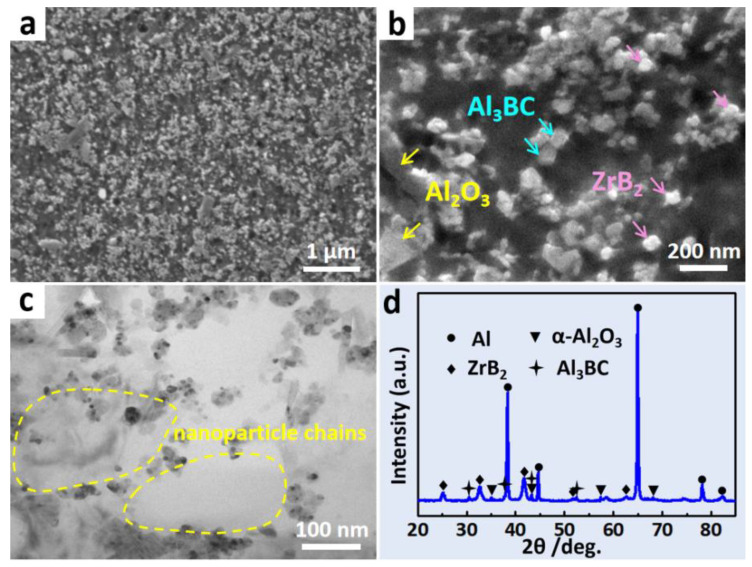
(**a**,**b**) The microstructure of the (ZrB_2_ + Al_3_BC + Al_2_O_3_)/Al composite analyzed by FESEM; (**c**) The TEM image showing the nanoparticle chains; (**d**) is the XRD pattern.

**Figure 3:**
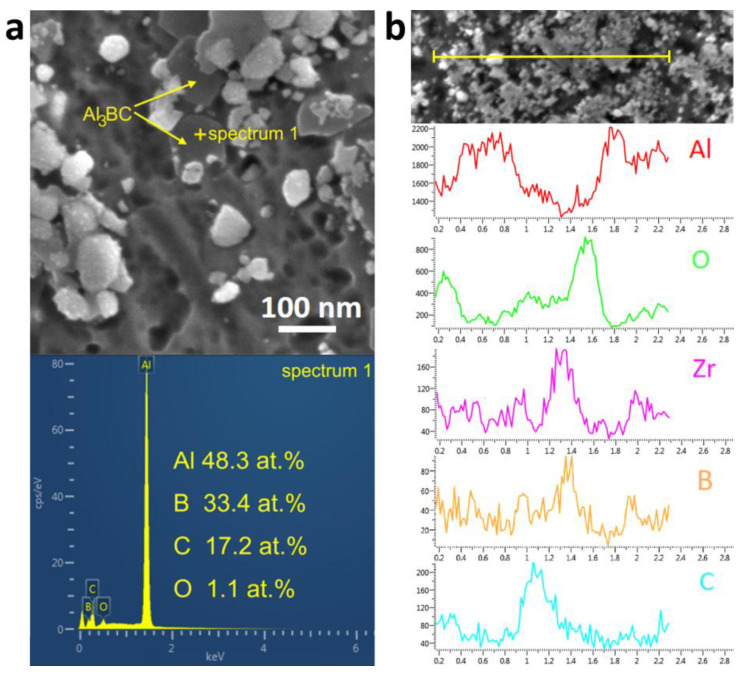
EDS result of the (ZrB_2_ + Al_3_BC + Al_2_O_3_)/Al composite: (**a**) Point analysis of the Al_3_BC; (**b**) Line scanning images of a typical area.

The microstructure of the (ZrB_2_ + Al_3_BC + Al_2_O_3_)/Al composite was first characterized by the FESEM, as shown in Figure 2a,b. It can be found that a large number of nano-sized particles distribute uniformly in the Al matrix under a relatively low magnification (Figure 2a). Figure 2b is a further magnified image, which shows that the Al_2_O_3_ particles exhibit an irregular shape with an average size of less than 200 nm, while the bright ZrB_2_ and dark Al_3_BC particles are hexagonal in shape with a size below 50 nm. The TEM result shows that these nanoparticles prefer to perform as particle chains, as marked in Figure 2c. Figure 2d is the corresponding XRD pattern of the composite, in which the diffraction peaks of ZrB_2_, Al_3_BC and Al_2_O_3_ can be clarified. It is worth noting that the full-width values at half maximum of the diffraction peaks are quite wide, which proves that these particles are relatively fine. To identify the elements in the reinforced particles, EDS analysis was performed. From the point detecting result (Figure 3a), it can be seen that in addition to the Al element, the B and C elements have mainly been detected, which may indicate that the dark hexagonal particles are Al_3_BC. Since the elements with light weight, e.g., B and C, can hardly be quantificationally detected by EDS, thus the line scanning analysis was further conducted for the (ZrB_2_ + Al_3_BC + Al_2_O_3_)/Al composite, as shown in Figure 3b. The corresponding peaks in the curves of Al, O, C, Zr and B may act as the evidence for above deduction, i.e., various nanoparticles have been successfully synthesized in the composite.

**Figure 4:**
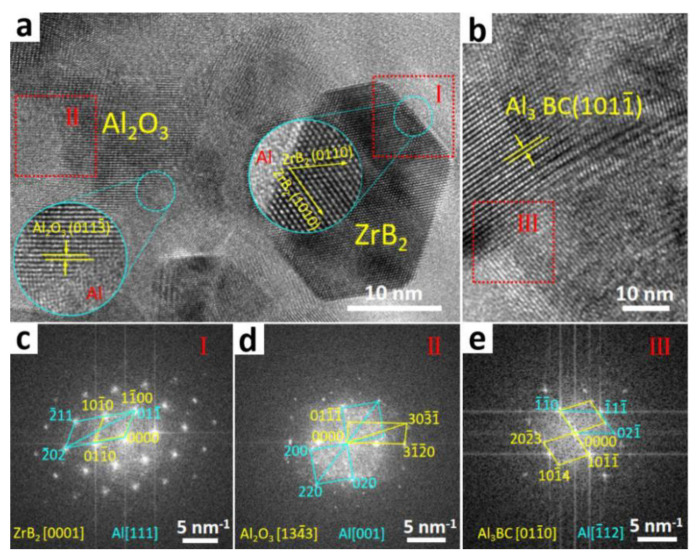
(**a**,**b**) The HRTEM images of the (ZrB_2_ + Al_3_BC + Al_2_O_3_)/Al composite; (**c**–**e**) The corresponding FFT patterns of the nanoparticles in (**a**,**b**).

In order to observe the interfacial structure of the Al matrix and nanoparticles, i.e., ZrB_2_, Al_3_BC and Al_2_O_3_, the HRTEM analysis of the composite has been carried out. Figure 4a,b are the high resolution lattice images of the nanoparticles whose morphology and size correspond well with the FESEM results. Judged by the magnified HRTEM image of the interface (marked by blue circles), it can be inferred that the nanoparticles and the α–Al grains are well bonded at the atomic scale by clear interfaces. To better present the crystal orientation of the relationships between nanoparticles and α–Al, the corresponding fast Fourier transform (FFT) patterns of the interfaces (red square marked by Ι, Ⅱ and Ⅲ) are displayed in Figure 4c–e, respectively. According to the FFT patterns, the paralleled crystal planes and directions between the particles and matrix can be achieved, e.g., [111]Al∥[0001]ZrB2, (01–1)_Al_*∥*(1–100)_ZrB2_.

**Figure 5:**
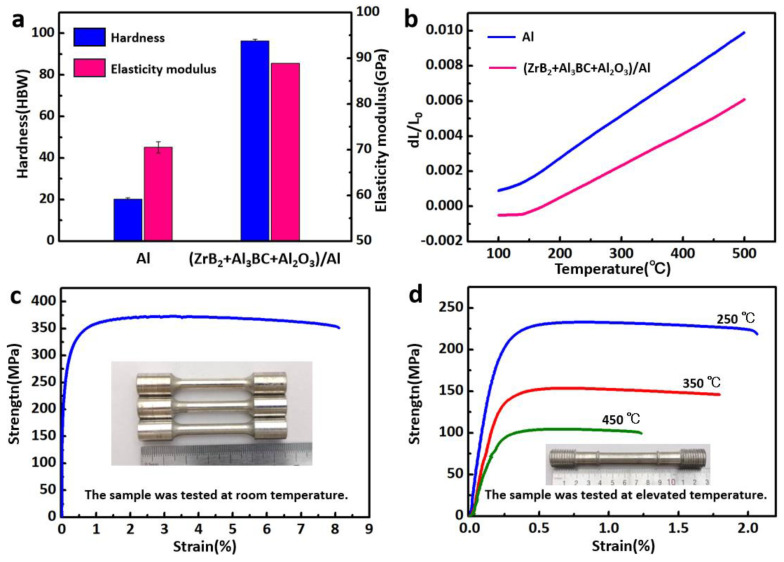
The hardness, elasticity modulus (**a**) and thermal expansion curves (**b**) of pure Al and the composite; (**c**,**d**) are the tensile curves of the composite tested at room temperature and elevated temperature.

The mechanical and thermal properties of the (ZrB_2_ + Al_3_BC + Al_2_O_3_)/Al composite are presented in Figure 5. It was found that compared with pure Al, the hardness and elasticity modulus of the composite have been improved by 375% and 26%, which are 96 HBW and 89 GPa, respectively. The thermal expansion curves of the composite and pure Al are shown in Figure 5b. The detected value of expansion ratio is relatively low, indicating that the composite exhibits excellent thermal stability. The tensile properties at room temperature and elevated temperature of the (ZrB_2_ + Al_3_BC + Al_2_O_3_)/Al composite are presented in Table 1, while the typical tensile curves are shown in Figure 5c,d, respectively. The ultimate tensile strength (UTS) and elongation (EI) of the composite are measured to be 371 MPa and 8.1% at 25 °C. With the increase in testing temperature, the UTS value decreases gradually. However, it should be noted that the composite can still maintain a relatively high UTS, e.g., 154 MPa at 350 °C and 104 MPa at 450 °C. It is also necessary to mention that the (ZrB_2_ + Al_3_BC + Al_2_O_3_)/Al composite has quite attractive comprehensive properties, when compared with our previous work and other composites [27,28,29,30]. Table 2 has listed the tensile properties of several composites which are reinforced with single particle or two kinds of particles. It can be seen that the (ZrB_2_ + Al_3_BC + Al_2_O_3_)/Al composite designed in this paper exhibits obvious advantages.

The attractive mechanical performance of the (ZrB_2_ + Al_3_BC + Al_2_O_3_)/Al composite is considered due to several reasons. Basically, the grain refinement effect caused by the in-situ formed particles cannot be neglected, since they can act as barriers to the growth of matrix grains during the synthesis procedure of the composite [31]. Except for the Hall–Petch strengthening effect, the Orowan strengthening mechanism and load transfer strengthening mechanism also play significant roles. Since amounts of nanoparticles locate in the Al matrix, dislocation movements occurring during the tensile procedure will be restrained by the particles, i.e., the Orowan strengthening mechanism [32]. Furthermore, the ZrB_2_, Al_3_BC and Al_2_O_3_ nanoparticles have good atomic bonding interfaces with the Al matrix as mentioned above, therefore, they can effectively transfer the stress from Al matrix to the nanoparticles during the tensile procedure. As a result, significant improvement of the tensile strength can be achieved through the so-called load transfer strengthening mechanism [33]. Besides, it should also be mentioned that since the nanoparticles are distributed as particle chains (Figure 1c), they may be more efficient acting as load bearing sites than uniformly distributed ones, which can be referred to in our previous work [34] and can be explained by the theory put forward by Hashin and Shtrikman [35].

**Figure 6:**
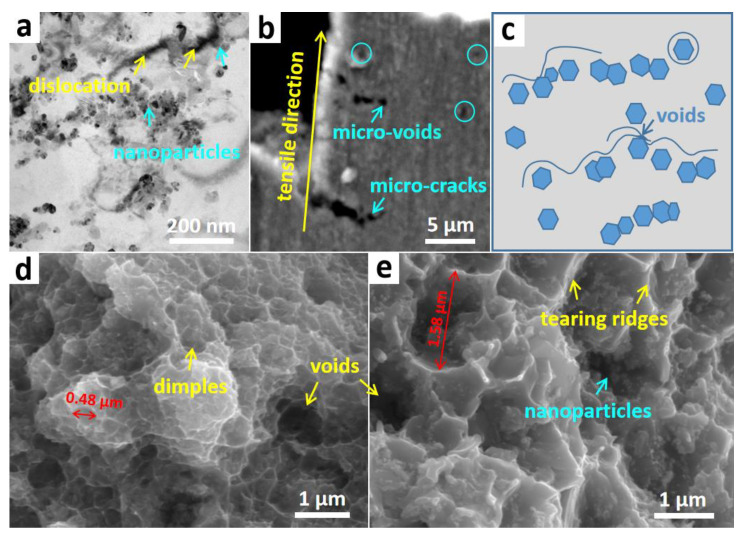
The characterization on the post-tensile sample of the composite: (**a**) is the TEM image; (**b**) is the FESEM result; (**c**) is the schematic showing the dislocation behavior in the tensile process; (**d**,**e**) The fracture surfaces of the composite after testing at 25 °C (**d**) and 350 °C (**e**).

To better comprehend the strengthening effect of nanoparticles and the failure mechanism of the composite, the post-tensile samples of the composite have been investigated. Figure 6a is the TEM result of the composite before fracture, in which the dislocations located between the Al grains and the nanoparticles are observed. During the tensile test procedure, more and more dislocations will concentrate around the particles when the stress is up to some extent, thus resulting in the formation of micro-voids, as shown in Figure 6b. Then, the micro-voids will connect to each other along the direction perpendicular to the tensile direction to produce micro-cracks, which finally leads to the fracture of the composite. For ease of understanding, the schematic is developed as shown in Figure 6c, clearly indicating the positive effect of ZrB_2_, Al_3_BC and Al_2_O_3_ particle chains in the composite. Figure 6d,e are the typical fracture surfaces of the composite after testing at 25 °C and 350 °C, respectively. Large numbers of distributed circular dimples can be found in the fracture surfaces, which further confirms the failure mechanism resulting from void growth, coalescence and failure. The statistic values of the dimple were calculated to be 0.48 μm and 1.58 μm for the fracture image under 25 °C and 350 °C tests, respectively (Figure 6d,e). Besides, compared with the fracture tested at 25 °C, the fracture tested at 350 °C is quite different. As presented in Figure 6e, a lot of sharp tearing ridges are exposed along with amounts of particles. As is widely known, the dynamic softening, i.e., rapid softening of the matrix, usually occurs at elevated temperatures, especially when the temperature is higher than 0.5 T_m_ (T_m_ is the melting point of Al, which is 660 °C) [36]. Therefore, it was deduced that the in–situ formed particles in this (ZrB_2_ + Al_3_BC + Al_2_O_3_)/Al composite act as a rigid skeleton and can hinder the occurrence of dynamic softening, thus resulting in attractive UTS at elevated temperatures.

On the whole, the experimental results above have confirmed that the designed composite exhibits attractive comprehensive performance. On the one hand, the developed composite in this paper may be applied in elevated temperature environments. On the other hand, this work proves that the idea of using different reinforcements to achieve comprehensive properties in one composite is feasible.

## 4. Conclusions

In this paper, a novel (ZrB_2_ + Al_3_BC + Al_2_O_3_)/Al composite has been in-situ synthesized, with the aim of exhibiting the respective advantages of the selected nano-sized particles. As a result, the expansion ratios of the composite were reduced significantly, and the modulus of the composite is 89 GPa. Besides, the ultimate tensile strengths at elevated temperatures are attractive, which are 154 MPa at 350 °C and 104 MPa at 450 °C. This paper may be referred to in the design of new Al composites with various simultaneous reinforcements.

## Figures and Tables

**Table 1:** The tensile properties of the composite at different temperatures.

Tensile Properties	Experiment Temperature (°C)			
	25	250	350	450
UTS (MPa)	371 ± 6	233 ± 10	154 ± 15	104 ± 5
EI (%)	8.1 ± 1.1	2.1 ± 0.5	1.8 ± 0.1	1.2 ± 0.1

**Table 2:** Comparison of the tensile properties of several composites.

Composites	UTS at 25 °C (MPa)	UTS at 350 °C (MPa)	Refs.
Al_2_O_3_/Al	268	121	[27]
ZrB_2_/Al	272	–	[28]
TiB_2_/Al–Si	298	96	[29]
(ZrAl_3_ + AlN)/Al	272	94	[30]
